# Growth of vanadium dioxide thin films on hexagonal boron nitride flakes as transferrable substrates

**DOI:** 10.1038/s41598-019-39091-8

**Published:** 2019-02-27

**Authors:** Shingo Genchi, Mahito Yamamoto, Koji Shigematsu, Shodai Aritomi, Ryo Nouchi, Teruo Kanki, Kenji Watanabe, Takashi Taniguchi, Yasukazu Murakami, Hidekazu Tanaka

**Affiliations:** 1 Institute of Scientific and Industrial Research, Osaka University, Ibaraki, Osaka 567-0047 Japan; 2 The Ultramicroscopy Research Center, Kyushu University, Fukuoka, 819-0395 Japan; 3 Department of Applied Quantum Physics and Nuclear Engineering, Faculty of Engineering, Kyushu University, Fukuoka, 819-0395 Japan; 4 Graduate School of Engineering, Osaka Prefecture University, Sakai, Osaka 599-8570 Japan; 5 JST PRESTO, Kawaguchi, Saitama 332-0012 Japan; 6 National Institute for Materials Science, Tsukuba, Ibaraki 305-0044 Japan

## Abstract

Vanadium dioxide (VO_2_) is an archetypal metal-insulator transition (MIT) material, which has been known for decades to show an orders-of-magnitude change in resistivity across the critical temperature of approximately 340 K. In recent years, VO_2_ has attracted increasing interest for electronic and photonic applications, along with advancement in thin film growth techniques. Previously, thin films of VO_2_ were commonly grown on rigid substrates such as crystalline oxides and bulk semiconductors, but the use of transferrable materials as the growth substrates can provide versatility in applications, including transparent and flexible devices. Here, we employ single-crystalline hexagonal boron nitride (hBN), which is an insulating layered material, as a substrate for VO_2_ thin film growth. VO_2_ thin films in the polycrystalline form are grown onto hBN thin flakes exfoliated onto silicon (Si) with a thermal oxide, with grains reaching up-to a micrometer in size. The VO_2_ grains on hBN are orientated preferentially with the (110) surface of the rutile structure, which is the most energetically favorable. The VO_2_ film on hBN shows a MIT at approximately 340 K, across which the resistivity changes by nearly three orders of magnitude, comparable to VO_2_ films grown on common substrates such as sapphire and titanium dioxide. The VO_2_/hBN stack can be picked up from the supporting Si and transferred onto arbitrary substrates, onto which VO_2_ thin films cannot be grown directly. Our results pave the way for new possibilities for practical and versatile applications of VO_2_ thin films in electronics and photonics.

## Introduction

In recent decades, transition metal oxides that undergo metal-insulator transitions (MITs) have attracted much attention as components for electronic and photonic devices. In particular, vanadium dioxide (VO_2_) has been of great interest for practical applications because the MIT for VO_2_ is induced near room temperature (~340 K)^1^. Additionally, the MIT leads to dramatic changes in the electrical resistivity (up-to five orders of magnitude in a single crystal)^2^ as well as optical transmittance (more than 50% in the infrared range)^3^. More importantly, thanks to advancements in thin film growth techniques, VO_2_ can be prepared consistently in thin film form, with properties comparable to those of the bulk counterpart^4^. In fact, the growth of high-quality VO_2_ thin films has been demonstrated even on the wafer scale, which is promising for practical use in electronics and photonics^5^.

Commonly, thin films of VO_2_ have been prepared on oxide substrates because a high-temperature oxygen (O_2_) treatment is often necessary for crystallization. Some of the most common substrates include aluminum oxide (Al_2_O_3_)^6^ and titanium dioxide (TiO_2_)^7^, on which VO_2_ films show a MIT with resistivity changing by up-to four orders of magnitude. Additionally, non-oxide materials have been employed as substrates for VO_2_ growth, such as silicon (Si)^8^, germanium^9^, and gallium nitride^10^. Even on such semiconducting compounds, VO_2_ films show reasonable MIT properties, opening the possibility for the realization of electronic and photonic VO_2_ devices compatible with existing semiconductor technology. On these rigid substrates, the fabrication of devices is, in principle, based on the top-down integration of other components onto VO_2_. However, if VO_2_ thin films can be prepared on thin transferable supports, the range of device applications is expected to be extended further, including for example, flexible devices.

Recently, with the progress of research on two-dimensional (2D) layered materials, layered materials have attracted interest as transferable substrates for growth of VO_2_ thin films. Recent studies have reported that VO_2_ thin films can be grown on chemical vapor deposited graphene on copper and that a VO_2_/graphene stack can be transferred onto a plastic film for use in flexible thermochromic windows^11,12^. Additionally, growth of VO_2_ on muscovite has been reported^13,14^. Thin films of muscovite could be peeled off from the bulk with the covering VO_2_^14^, hence promising flexible device applications. Although these results suggest the utility of layered materials for the VO_2_ growth substrate, their potential applications may be limited in number because graphene shows an extremely high electrical conductivity, while muscovite is mechanically relatively fragile. Therefore, the search for alternative layered materials with insulating properties as well as mechanical strength is necessary for realizing more versatile applications.

**Figure 1:**
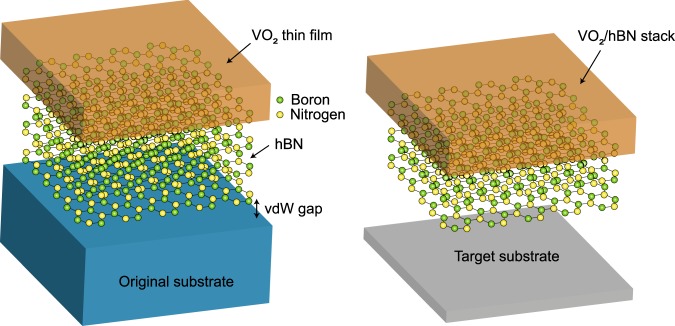
Schematic illustration of the growth of the VO_2_ thin film on a hBN flake supported on a substrate (left). Due to the weak van der Waals (vdW) interaction between hBN and the substrate, the stack of VO_2_ and hBN is expected to be transferred from the original substrate onto a target substrate of any material and geometry (right).

Here, we demonstrate the growth of VO_2_ thin films on crystalline hexagonal boron nitride (hBN), which is a layered material consisting of honeycomb lattices with nitrogen and boron atoms arranged at the inequivalent triangular sites. Hexagonal BN is electrically insulating even at nanometer thicknesses^15,16^, due to a wide bandgap energy close to 6 eV^17,18^. Ultrathin hBN has a large Young’s modulus of ~0.86 TPa and a fracture strength of ~70 GPa^19^, comparable to those of diamond. In addition to these exceptional electronic and mechanical properties, hBN is inert to O_2_ exposure even at 500 °C^20,21^, making it a promising choice for use as an oxide growth substrate. We observed that VO_2_ thin films could be grown on hBN with crystallinity and that such films underwent a MIT at approximately 340 K. Across the MIT, the resistivity was observed to change by nearly three orders of magnitude, which is comparable to that found for VO_2_ thin films grown on common substrates^6–10^. More importantly, we found that the stack of VO_2_ and hBN could be picked up from an original substrate, and, then, transferred onto another target substrate of any material and geometry, as schematically depicted in Fig. 1. These results have important implications for the realization of a variety of electronic and photonic devices based on VO_2_/hBN stacks.

## Results and Discussion

**Figure 2:**
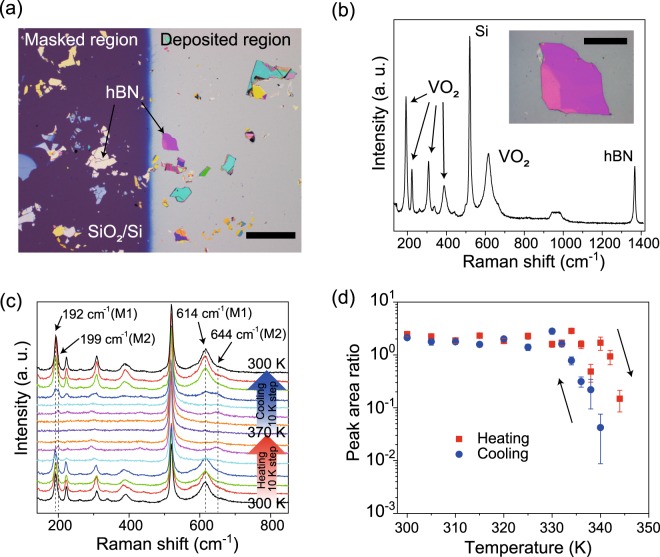
(**a**) Optical image of hBN flakes exfoliated onto a SiO_2_/Si substrate after the growth of VO_2_. The left purple region shows the SiO_2_/Si surface, which was masked during the growth, while the deposited region is colored in gray. The scale bar is 250 μm. (**b**) Raman spectrum measured for the VO_2_ thin film grown on hBN on SiO_2_/Si at 300 K. The inset shows the optical image of the VO_2_/hBN stack. The scale bar is 50 μm. (**c**) Temperature-dependent Raman spectra measured at VO_2_ on hBN. The measurement temperature was increased from 300 to 370 K and, subsequently, decreased from 370 to 300 K with a 10 K step. (**d**) The area ratio of the VO_2_ peak at 614 or 644 cm^−1^ to the Si peak at 520 cm^−1^ as a function of temperature in the heating (red square dot) and cooling (blue circular dot) processes.

For the VO_2_ growth substrates, we used thin flakes of high-purity hBN single crystals, which were synthesized by the method described in ref.^22^. Using adhesive tape, thin flakes of hBN were mechanically exfoliated from bulk single crystals onto Si substrates with 285 nm-thick silicon dioxide (SiO_2_) layers, leading to the (0001) surface facing upward. The exfoliated hBN flakes have atomically flat surfaces with few defects^15,16^. Following an O_2_ treatment at 500 °C in ambient pressure for 3.5 hours to remove the adhesive tape residue^20^, thin films of VO_2_ were grown onto hBN by pulsed laser deposition (PLD, see Methods for more details). Figure 2a is a typical optical image of the hBN flakes supported on SiO_2_/Si after the growth of a VO_2_ thin film. The lateral sizes of the hBN flakes exfoliated on SiO_2_ range up-to hundreds of micrometers in length. By measuring the height difference between the region covered with VO_2_ and the substrate by using atomic force microscopy (AFM), the thickness of the deposited film was determined to be ~26.9 nm. Below, by using cross-sectional scanning transmission electron microscopy (STEM), we find that the thicknesses of films supported on SiO_2_ and hBN are comparable to each other. First, the crystallinity of VO_2_ on hBN was characterized using Raman spectroscopy. Figure 2b is a Raman spectrum obtained for VO_2_ grown on a 226 nm-thick hBN flake at 300 K (see the inset for the optical image of the VO_2_/hBN stack). In addition to the peaks for Si and hBN at 520 and 1367 cm^−1^ ^23,24^, prominent Raman peaks from the vibration modes of monoclinic VO_2_ were observed at 192, 222, 307, 389, and 614 cm^−1^ ^25^, indicating the growth of the VO_2_ crystal on hBN.

Additionally, we investigated the phase transition properties of the VO_2_ films grown on hBN from temperature-dependent Raman spectroscopy measurements. As shown in Fig. 2c, the Raman peaks for VO_2_ on a hBN flake were diminished above 340 K when heated up from 300 K but appeared when cooled down from 370 K below 330 K. The observations imply that VO_2_ on hBN undergoes a structural phase transition between the monoclinic (which is commonly called the “M1” phase) and tetragonal structures at a temperature of approximately 340 K^26^. We note that the Raman peaks for VO_2_ with a tetragonal structure are absent, as reported previously^25,26^. Noteworthy, near the transition temperature, strong peaks were observed at 199 and 644 cm^−1^, rather than 192 and 614 cm^−1^ (see Fig. 2c). The Raman peaks are characteristics of the monoclinic “M2” phase of VO_2_, which is the metastable transient structure between the monoclinic M1 and tetragonal structures and can be stabilized by doping^27,28^ or applying strain along the [110] direction of the rutile structure^29,30^. Although we cannot completely exclude the possibility of unintentional doping in VO_2_ without a detailed chemical analysis, since no dopants were added during growth, the stabilization of the M2 phase in VO_2_ on hBN is most likely due to strain along the [110] direction. Below, from cross-sectional STEM measurements, the VO_2_ thin film is observed to be grown preferentially with the (110) orientation. Therefore, VO_2_ is supposed to be compressively strained isotropically in the in-plane direction on hBN, leading to tensile strain along the [110] direction. Such interfacial strains have been observed in thin films grown on layered materials and are explained to be due to the dipole-dipole interactions^31^. Further detailed measurements with, for example, synchrotron X-ray diffraction spectroscopy will be needed to identify the cause of the stabilization of the M2 phase in VO_2_ grown on hBN.

The transition temperature estimated from the Raman spectroscopy measurements in Fig. 2c is supposed to be lower than the exact transition temperature because the actual temperature of VO_2_ is higher than the set value due to laser heating during the measurements. To further explore the critical temperature for the structural phase transition, we collected the Raman spectra for VO_2_ using a much smaller laser intensity than that used in Fig. 2c (see Methods). Figure 2d shows the area ratio for the VO_2_ peak at 614 or 644 cm^−1^ to the Si peak at 520 cm^−1^ as a function of temperature for the heating and cooling processes. We found that the Raman peak area ratio was reduced abruptly at 344 K in the heating process, but, in the cooling process, increased largely at 340 K. We note that the actual critical temperature for the structural phase transition of VO_2_ could be still higher than the obtained values because the influence of laser heating cannot be completely ruled out. The observations suggest that the structural phase transition is induced with hysteresis. The magnitude of hysteresis is as small as that reported in the Raman spectroscopy measurement for single-crystalline VO_2_^32^, implying that the VO_2_ film on hBN is a single crystal or polycrystalline with grain sizes comparable to the laser spot size of the Raman spectroscopy measurement of ~1 μm.

**Figure 3:**
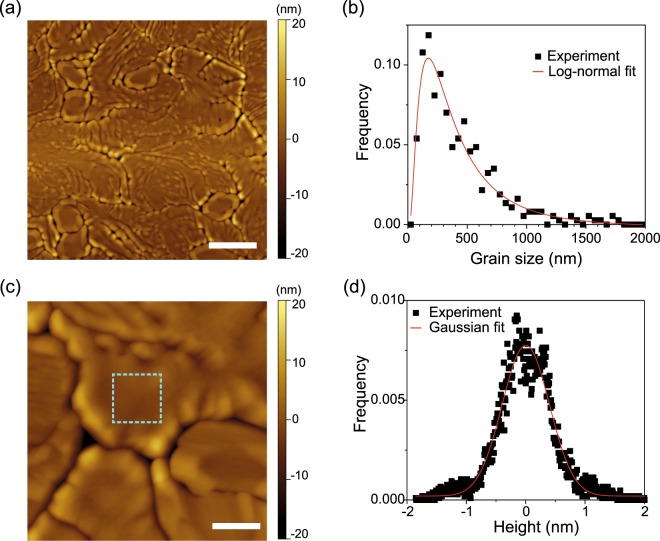
(**a**) AFM topographic image of VO_2_ grown on hBN. The scale bar is 1 μm. (**b**) The distribution of the grain size of VO_2_ (black square dots). The red curve is a logarithmic-normal fit. (**c**) Magnified AFM image of VO_2_ on hBN. The scale bar is 200 nm. (**d**) The height histogram for the area surrounded by the dashed light blue line in (**c**). The red solid curve is a Gaussian fit with a standard deviation of 0.4 nm.

Next, we investigated the morphology of VO_2_ thin films grown on hBN using AFM. Figure 3a shows an AFM image of a VO_2_ thin film grown on a hBN flake collected at room temperature. The VO_2_ thin film on hBN was observed to be composed of grains with meandering boundaries, thus suggesting a polycrystalline nature, at least, in the in-plane direction. To estimate the characteristic grain size of VO_2_ on hBN, we measured the spacing between grain boundaries over the AFM image. Figure 3b shows the distribution of the VO_2_ grain sizes on hBN. As often seen in polycrystalline films^33^, the grain size distribution can be well fitted with a logarithmic-normal function, with the mean size determined to be 490 ± 60 nm in length. Although we cannot be certain that each grain is single-crystalline, the observed grain size as large as micrometers is consistent with the expectation from the Raman spectroscopy measurements. The grain size of VO_2_ on hBN is one order of magnitude larger than that of polycrystalline VO_2_ on Al_2_O_3_(0001)^34^, where small grains are formed as a result of the ease of the large lattice mismatch strain (see Supplementary Information for the AFM image). Therefore, compared with the VO_2_ films grown on Al_2_O_3_, VO_2_ on hBN is supposed to be strained slightly during growth. While the grains of VO_2_ on hBN were found to upheave by ~10 nm near the boundaries, which is likely due to the effect of grain boundary grooving^35^, each grain has a relatively flat surface in the middle, with a roughness of <0.4 nm, as shown in the AFM image and the height distribution in Fig. 3c,d. The observation of an atomically flat surface may imply single crystallinity for each VO_2_ grains on hBN.

**Figure 4:**
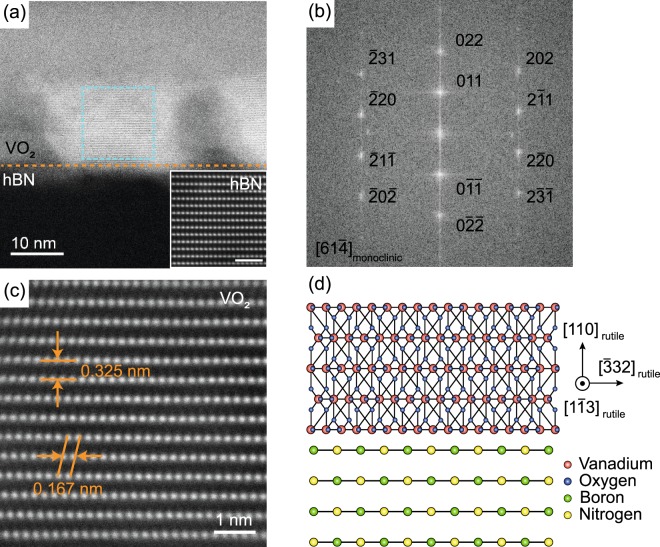
(**a**) Cross-sectional STEM image of the VO_2_/hBN stack. The orange dashed line indicates the interface between VO_2_ and hBN. The inset shows an atomically resolved STEM image of hBN. The scale bar in the inset is 1 nm. (**b**) FFT image of the VO_2_ region surrounded by the dashed light blue line in (**a**). The Miller indices of the spots are indicated by choosing the [61$\bar{4}$] of the monoclinic structure as the projection direction. (**c**) Atomically resolved STEM image of VO_2_. (**d**) Schematic illustration of VO_2_ grown on hBN with the orientation of the [110] direction of the rutile structure. The relative atomic position of VO_2_ compared to hBN and the atomic structure of VO_2_ at the interface do not necessary reflect the actual configuration.

To examine the crystallinity of the VO_2_ grains on hBN at the atomic scale, we employed high-angle annular dark-field STEM (HAADF-STEM. See Methods for the details)^36^. The specimens for the STEM measurements were prepared by cutting a VO_2_/hBN stack by focused ion beam milling. Figure 4a is a cross-sectional STEM image of the VO_2_/hBN stack obtained at room temperature. While the crystal structure of hBN cannot be well resolved from this viewing angle, the lattice fringe is clearly seen for the VO_2_ region over the sample. The inset in Fig. 4a shows the atomically resolved STEM image of hBN obtained from a different viewing angle. The darker regions imply that other VO_2_ grains with different orientations are present along the projection direction in the specimen. The crystallinity of the VO_2_ grain is confirmed from the sharp spots observed in the fast Fourier transformation (FFT) image extracted from the area surrounded by the dashed light blue line in Fig. 4a (see Fig. 4b for the FFT image; the assignments for the spots will be discussed in the following). To determine the crystallographic orientation of the grain, we obtained a high-resolution STEM image of VO_2_, as shown in Fig. 4c. Vanadium atoms are observed to be arranged with an out-of-plane spacing of 0.325 nm, with an in-plane spacing of 0.167 nm. We note that the out-of-plane lattice spacing of VO_2_ is determined by calibration with a known interlayer spacing of hBN of 0.333 nm (see the inset of Fig. 4a). Since the monoclinic and rutile VO_2_ show <1% difference in the spacing between neighboring vanadium atoms, we characterize the crystallographic orientation of the VO_2_ grain based on the rutile structure, for simplicity. The out-of-plane lattice spacing of 0.325 nm could be assigned as the spacing between the (110) plane of the rutile VO_2_, which is 0.322 nm for the bulk. Therefore, the VO_2_ crystal on hBN is strained by 1% along the [110] direction of the rutile structure, while the in-plane strain is estimated to be 0.3% by assuming the Poisson’s ratio to be 0.3^37^. Such magnitude of tensile strain along the [110] direction is large enough to stabilize the M2 phase in VO_2_ near the transition temperature^29^. Given the observed in-plane spacing, the projection direction can be, therefore, identified along the [1$\bar{1}$3] direction of the rutile structure (see Fig. 4d for a schematic illustration). In the presence of four crystallographic variants, there are four candidate directions in the monoclinic VO_2_ (i.e., [61$\bar{2}$], [6 $\bar{1}\bar{2}$], [6 $\bar{1}\bar{4}$], and [61$\bar{4}$]) to which the rutile [1$\bar{1}$3] direction can be transformed. Therefore, among the variants taking the [61$\bar{4}$] orientation for the projection, for example, the spots in the FFT image can be assigned, as shown in Fig. 4b.

From the STEM image of the other specimen, we observed that the VO_2_ grain is, again, orientated along the [110] direction of the rutile structure (see Supplementary Information). Therefore, VO_2_ is presumably grown on hBN, most preferentially with the (110) orientation of the rutile structure. We note that VO_2_ is grown in the rutile structure since the growth temperature is 450 °C^4^. A density functional theory calculation has shown that the (110) surface is the most energetically favorable among the low Miller index planes of VO_2_^38^, and, indeed, growth of polycrystalline VO_2_ films with the (110) orientation has been observed on Si and quartz^30^. Therefore, the preferential growth of the VO_2_ film with the (110) orientation on hBN is legitimate because the hBN surface has no dangling bonds, and hence, only weak van der Waals interactions are expected to act at the interface (see Fig. 4d). Such so called “van der Waal epitaxy” of VO_2_ has been reported previously on a muscovite(0001) substrate, but the growth orientation is not [110] but in the [010] direction^14^. This difference might be because the layers of muscovite are negatively charged^39^ in contrast with hBN, and the electrostatic interactions, in addition to the van der Waals interactions, play a role at the growth interface. As shown in Supplementary Fig. S2, we also observed Moiré patterns in the STEM images of VO_2_, which are most likely produced by superposition in the multiple grains and/or the monoclinic variants in the VO_2_ film (see Fig. 3c for the AFM image of the VO_2_ film with grains). To settle the growth orientations, therefore, further detailed analysis for the Moiré patterns as well as plan-view TEM measurements will be necessary.

**Figure 5:**
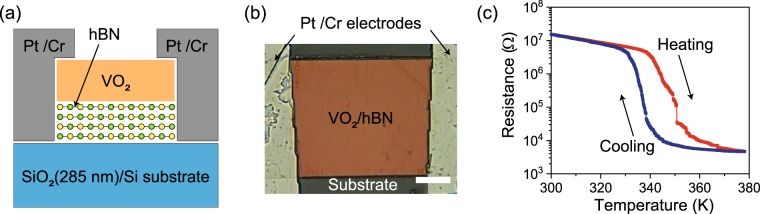
(**a**) Schematic and (**b**) optical images of the VO_2_/hBN stack contacted with the Pt/Cr electrodes for the electrical measurements. The scale bar in the optical image is 10 μm. (**c**) Resistance-temperature characteristic of the VO_2_/hBN stack. The electrical resistance of VO_2_ was measured from 300 to 378 K (red curve) and, subsequently, from 378 to 300 K (blue curve).

From the Raman spectroscopy measurements, we found that VO_2_ films grown on hBN underwent a structural phase transition at approximately 340 K (see Fig. 2d). Here, we investigate if the structural phase transition in VO_2_ on hBN accompanies an electronic phase transition. Figure 5a,b show schematic and optical images of the VO_2_/hBN stack with the platinum (Pt)/chromium (Cr) contact electrodes used for the electrical measurement. See Methods for details of the electrical measurements. The length and width of VO_2_ between the electrodes was 41 and 37 μm, while the thickness of VO_2_ and supporting hBN was estimated to be 40 and 90 nm, respectively. The resistance of VO_2_ was measured in a two-probe configuration at temperatures ranging between 300 and 378 K. Figure 5c shows the resistance-temperature characteristics for VO_2_ on hBN measured in the heating (red curve) and cooling (blue curve) processes. At 300 K, VO_2_ shows a resistance of <10^7^ Ω; however, the resistance was observed to be largely reduced with increasing temperature above ~344 K. Ultimately at 378 K, the resistance was decreased down to the order of 10^3^ Ω. Alternatively, when the VO_2_ film was cooled down, the resistance showed a large increase below ~336 K. These observations clearly indicated that the MIT for VO_2_ on hBN was thermally induced at approximately 340 K with hysteresis, similar to polycrystalline VO_2_ films grown on other substrates such as Al_2_O_3_(0001)^6^. The magnitude of the resistance change across the MIT is comparable to that observed in VO_2_ grown on common substrates^6–10^, emphasizing the usefulness of hBN as the VO_2_ growth substrate.

We observed several resistance jumps, particularly during the heating process for the resistance-temperature curve in Fig. 5c. Similar behaviors were previously observed for the resistance-temperature characteristics of VO_2_ thin films grown on Al_2_O_3_(10$\bar{1}$0)^40^ and TiO_2_(001)^41^, which were explained by the effects of the crystal domain sizes relative to the spacing between the measurement electrodes. Thin films of VO_2_ commonly consist of crystal domains, with each domain having a slightly different critical temperature for the MIT. As a result, when the temperature-dependent electrical measurement is conducted for a VO_2_ thin film, the resistance does not change thoroughly at a unique temperature, but gradually with temperature in a percolating manner. However, if the spacing between the measurement electrodes is narrowed down to the scale of the domain size, the formation of single percolation paths between the electrodes becomes marked in the resistance change, resulting in step-like behaviors in the resistance-temperature characteristics. The typical domain size for polycrystalline VO_2_ on Al_2_O_3_ was measured to be tens of nanometers, and, hence, a nanometer-scale electrode spacing was necessary to observe such resistance jumps^40^. For single-crystalline VO_2_ on TiO_2_(001), the domain is regulated by the cracks formed in the film, which is as large as micrometers in size^41^. Therefore, resistance jumps were observed even for an electrode spacing of 50 μm. The resistance jumps in VO_2_ on hBN were observed with a 41 μm-long electrode spacing, implying that the characteristic domain size is on the order of hundreds of nanometers or even micrometers, which is consistent with the observed grain sizes in Fig. 3. Therefore, with decreasing electrode spacing close to the domain size, the resistance change for VO_2_ on hBN is expected to be more step-like, and, ultimately, to exhibit a single step across the MIT, as observed in single-crystalline VO_2_^2^.

**Figure 6:**
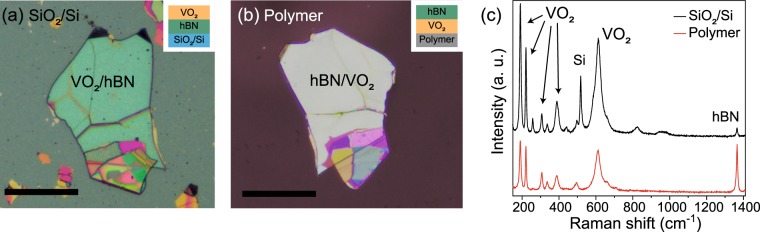
Optical images of the VO_2_/hBN stack on (**a**) the SiO_2_/Si substrate and (**b**) transferred onto the polymer film. The scale bars are 50 μm. The insets in (**a**,**b**) show schematic illustrations of the stacking structure. (**c**) Raman spectra for the VO_2_/hBN stack before (black curve) and after (red curve) the transfer process. The prominent peaks due to the vibrational modes of VO_2_ can be seen even after the transfer.

Finally, although the actual fabrication of devices is beyond the scope of this paper, we performed a transfer of the VO_2_/hBN stack for device applications. Figure 6a,b show optical images of the VO_2_/hBN stack before and after the transfer process. See Methods for details of the pick-up and transfer process. By this process, the stacks were uniformly transferred from the substrate to the polymer film, as can be confirmed from the Raman spectra for VO_2_ in Fig. 6c. The polymer film has an elastomeric property and has been previously commonly used for stretchable electronics^42^. Therefore, the VO_2_/hBN stacks supported on the polymer could form the basis of electronic and optical switching devices with flexibility, bendability, and stretchability. To further validate the transferability of the VO_2_/hBN stacks, we transferred the stacks from the polymer films to other substrates such as gold, glass slide and paper, where the direct growth of a high-quality VO_2_ thin film is challenging (see Supplementary Information for optical images of VO_2_/hBN transferred onto these substrates). The Raman spectroscopy measurements confirmed uniform crystallinity for the transferred VO_2_, suggesting the utility of hBN as a transferrable substrate.

## Conclusion

We demonstrated growth of VO_2_ crystals on an insulating layered material, hBN. The VO_2_ film is polycrystalline, but the mean grain size was observed to reach up-to micrometers. The VO_2_ thin film is grown preferentially with the (110) orientation, which is predicted by the DFT calculation to be the most energetically stable^38^. Thin films of VO_2_ on hBN undergo a MIT at approximately 340 K, with the electrical resistance changing by nearly three orders of magnitude, showing high quality comparable to those of VO_2_ films grown on common substrates^6–10^. Most importantly, VO_2_ thin films could be transferred onto arbitrary surfaces with the supporting hBN; therefore, a broad spectrum of applications in electronics and photonics can be envisioned. For example, by transfer of the VO_2_/hBN stack onto an elastomer, flexible and stretchable devices could be fabricated. Thin flakes of hBN show good insulating and dielectric properties and also show atomically flat surfaces^43^. Therefore, the VO_2_/hBN heterostructure could form the basis of a “Mott transistor”^44^, where the MIT is controlled by gating. Additionally, the VO_2_/hBN heterostructure could behave as a reconfigurable hyperbolic metasurface^45^, with great potential for applications in nanophotonic devices. Moreover, due to an inert surface with no dangling bonds, hBN is expected to have a wide degree of freedom as a substrate for oxide thin film growth.

## Methods

### Growth of VO_2_ thin films on hBN flakes

Thin films of VO_2_ were grown onto hBN flakes by PLD using an argon fluoride excimer laser with a wavelength of 193 nm under an oxygen pressure of 0.95 Pa at a substrate temperature of 450 °C. The repetition rate was 2 Hz and the growth time was 5 h. A vanadium pentoxide pellet was used for the target.

### Characterization

The Raman spectroscopy measurements were conducted using a commercial system (Raman Touch, Nanophoton) with a solid state laser operating at a wavelength of 532 nm at 300–380 K. The temperature was controlled by placing a sample onto a thermally conductive plate connected to a Peltier heating/cooling device. The grating size was 1200 lines/cm. The laser spot size was ~ 1 μm and the laser power was set to be either 0.2 or 1 mW. The AFM measurements were conducted at room temperature in the ambient with the dynamic force mode using Si cantilevers (SPA-300HV, Hitachi High-Tech Science). The HAADF-STEM measurements were performed using a JEOL JEM-ARM200F ACCELARM operated at an acceleration voltage of 200 kV. The probe semi-angle was 18 mrad. The probe current was 9 pA. The angular detection range of the HAADF detector for the scattered electrons was 50–150 mrad.

### Electrical measurements

The stacks of VO_2_/hBN were etched into microwires by photolithography and reactive ion etching (RIE) under a mixture of O_2_ and sulfur hexafluoride gases. The contacting electrodes were prepared by photolithography and sputtering deposition of Cr (5 nm) and Pt (200 nm). Electrical measurements were conducted in air using a source measurement unit (2635A, Keithley) at temperatures ranging from 300 to 378 K, which was controlled by the Peltier heating/cooling system.

### Pick-up and transfer process

To pick-up the stack of VO_2_ and hBN, we used commercially available polymer films (PF Gel-Film, Gel-Pak)^46^ treated with water vapor, which show enhanced adhesion^47^. The polymer film covered with a thin water layer was placed onto the SiO_2_/Si substrate with the VO_2_/hBN stacks, and, then, was slowly peeled off from the substrate. The polymer films with the stacks were placed onto the target substrates using a transfer system (IZU-NNHA, Izumi-Tech), which was equipped with an optical microscope and micromanipulator.

## Supplementary information

Supplementary Information

## Data Availability

All data generated or analyzed during this study are included in this published article and its Supplementary Information file.
