# Supplementary material for: Growth of vanadium dioxide thin films on hexagonal boron nitride flakes as transferrable substrates
Source: Sci Rep. 2019 Feb 27;9:2857. doi: 10.1038/s41598-019-39091-8 (PMC6393539; doi:10.1038/s41598-019-39091-8)
Supplement: Supplementary file 1 — Supplementary Information [file 41598_2019_39091_MOESM1_ESM.pdf]

Supplementary Information for

Growth of vanadium dioxide thin films on hexagonal boron nitride flakes as transferrable substrates

Shingo Genchi[1], Mahito Yamamoto[1,*], Koji Shigematsu[2], Shodai Aritomi[3], Ryo Nouchi[4, 5], Teruo Kanki[1], Kenji Watanabe[6], Takashi Taniguchi[6], Yasukazu Murakami[2, 3] & Hidekazu Tanaka[1,*]

[1]Institute of Scientific and Industrial Research, Osaka University, Ibaraki, Osaka 567-0047, Japan

[2]The Ultramicroscopy Research Center, Kyushu University, Fukuoka 819-0395, Japan.

[3]Department of Applied Quantum Physics and Nuclear Engineering, Faculty of Engineering, Kyushu University, Fukuoka 819-0395, Japan.

[4]Graduate School of Engineering, Osaka Prefecture University, Sakai, Osaka 599-8570, Japan

[5]JST PRESTO, Kawaguchi, Saitama 332-0012, Japan

[6]National Institute for Materials Science, Tsukuba, Ibaraki 305-0044, Japan

[*]Correspondence and requests for materials should be addressed to M.Y. (mahito.yamamoto@sanken.osaka-u.ac.jp) or H.T. (h-tanaka@sanken.osaka-u.ac.jp)

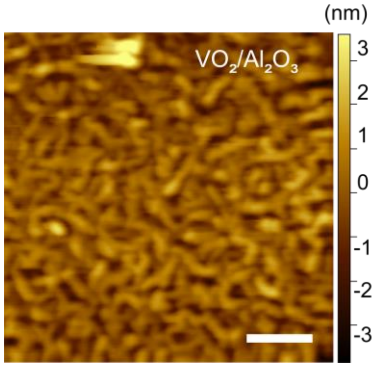

Figure S1. AFM image of VO_2 grown on Al_2O_3(0001) by PLD, showing the formation of the nanometer-scale grains. The scale bar is 200 nm. The grain size is estimated to be 20-30 nm in length.

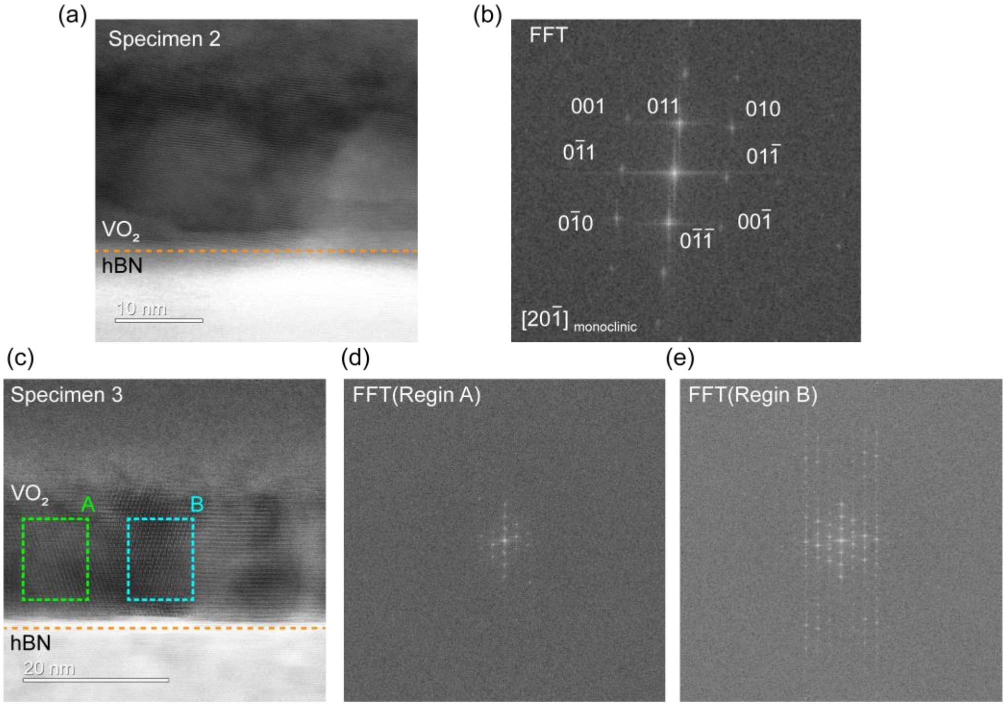

Figure S2. (**a**) Cross-sectional STEM image of the VO_2/hBN stack and (**b**) the FFT image extracted from the entire VO_2 region. The VO_2 film is observed to be oriented along the [110] direction of the rutile structure. The STEM image can be identified to be the projection of the [001] direction of the rutile structure, corresponding to the [20$\bar{1}$] projection of the monoclinic structure. (**c**) Cross-sectional STEM image of the VO_2/hBN stack obtained from the different specimen from one in (**a**). (**d and e**) FFT images obtained from the regions surrounded by the dashed green (Region A) and light-blue (Region B) lines the STEM image in (**c**). The Moiré patterns are clearly observed in the regions A and B.

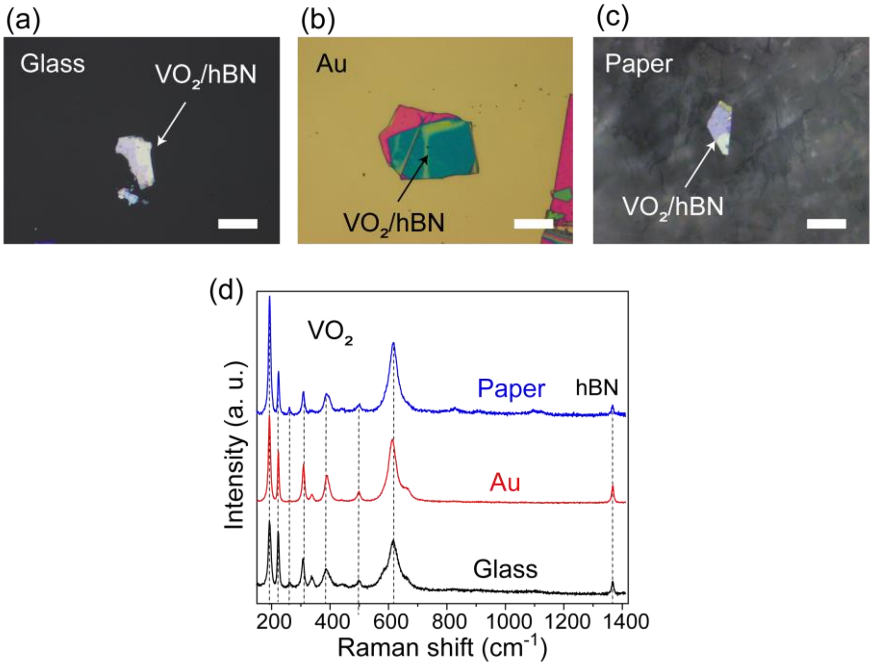

Figure S3. Optical images of the VO_2/hBN stacks transferred onto (**a**) a glass slide, (**b**) a gold (Au) film, and (**c**) a weighing paper. The scale bars are 10 μm. (**d**) Raman spectra of the stacks on the glass slide (black curve), Au (red curve), and the paper (blue curve). The prominent peaks of VO_2 are observed for all the samples, suggesting that the crystallinity of the VO_2 films are retained after the transfer process.
